# Supplementary material for: Redirection of ambient light improves predator detection in a diurnal fish
Source: Proc Biol Sci. 2020 Jan 22;287(1919):20192292. doi: 10.1098/rspb.2019.2292 (PMC7015323; doi:10.1098/rspb.2019.2292)
Supplement: Electronic Supplement with figures S1-S4 and tables S1-S4 [file rspb20192292supp1.docx]

Matteo Santon, Pierre-Paul Bitton, Jasha Dehm,
Roland Fritsch, Ulrike K. Harant, Nils Anthes, Nico K. Michiels

Redirection of ambient light improves predator detection in a diurnal fish: Electronic supplementary materials (ESM)

Proceedings of the Royal Society B

doi: 10.1098/rspb.2019.2292

Corresponding author: [nico.michiels@uni-tuebingen.de](mailto:nico.michiels@uni-tuebingen.de)

**1. General pictures Page**

- Figure S1 2

**2. Details of the statistical analyses**

- Methods S1 3
- Figure S2 4
- Table S1 5
- Table S2 6-7
- Table S3 7

**3. Details of the visual modelling**

- Methods S2 8
- Figure S3 10
- Figure S4 11
- Table S4 12

**4. References** (ESM) 13

**1. General pictures**


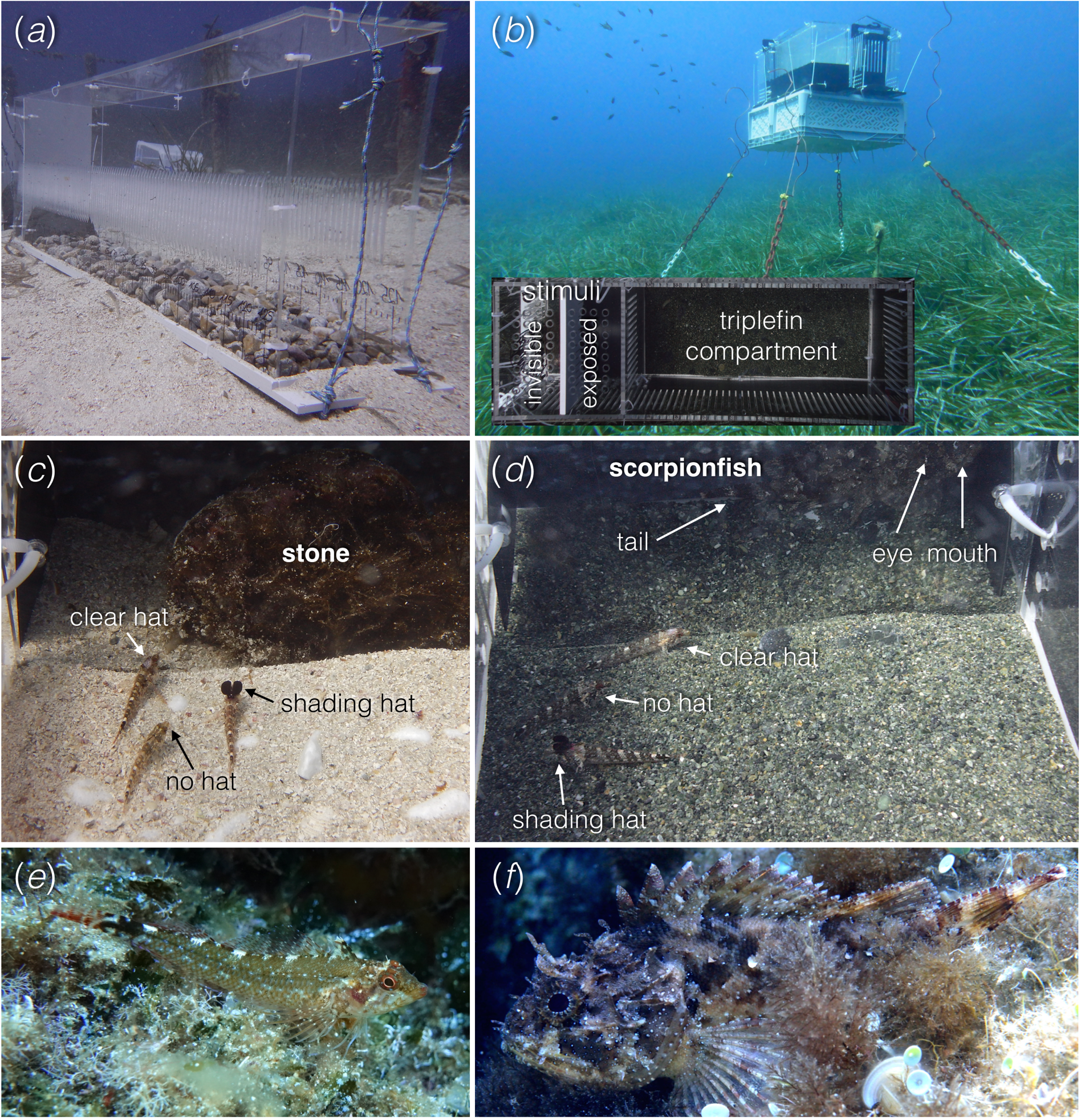


**Figure S1: Field experimental setups and studied species.** (*a*) Prototype of large tanks used for the first field experiment at 15 m depth. The final design had white blinds along the lower sides (compare black blinds in (*b*)) and the substrate was covered in white sand, as in (*c*). (*b*) For the second field experiment at 10 m depth, two smaller tanks were mounted on a float and fitted with black blinds and a dark sandy substrate. The insert shows a top view of the triplefin compartment and the compartments in which the stimulus (stone or scorpionfish) were either exposed to the triplefins (right), or kept out of view (left). Water flow between all compartments and the surrounding water was assured through narrow slits in the top half of all sides and inner separators. (*c*) A scene from a triplet of triplefins inspecting a stone in the display compartment on the bright sand in the setup at 15 m depth. (*d*) Same, but on the dark sand as used in the second field experiment, and with a scorpionfish as visual stimulus. (*e*) Adult *Tripterygion delaisi* and (*f*) *Scorpaena porcus* on natural substrate in the field in STARESO, Corsica, at about 6 m depth. See Methods for tank dimensions.

**2. Details of the statistical analyses**

***Methods S1***

### Triplet as random factor and model selection

In the first two statistical models, the initial random component contained *triplet ID* with random slopes over *hat treatment*. This accounts for the repeated measurements of each triplet and captures variation arising from different hat treatment responses among triplets [1]. Random slopes were uninformative and subsequently removed. In the third model, the random component included *triplefin ID*, *tank ID* and *cohort*. We then performed backward model selection using the Akaike Information Criterion (AIC) to identify the best-fitting model with the smallest number of covariates [2]. We only report the reduced final models and provide proxies for their overall goodness-of-fit (marginal and conditional *R*^2^) using the R package piecewiseSEM [3]. The marginal *R*^2^ expresses the proportion of variation explained by the model considering fixed factors only, whereas the conditional *R*^2^ expresses the same including the random factors [4]. We used Wald *z*-tests to assess the significance of fixed effects. To explore significant interactions between *stimulus* and *hat treatment*, we implemented new models within the two levels of *stimulus* (stone or scorpionfish). Model assumptions were validated by plotting residuals versus fitted values and each covariate present in the full, non-reduced model [5].

**
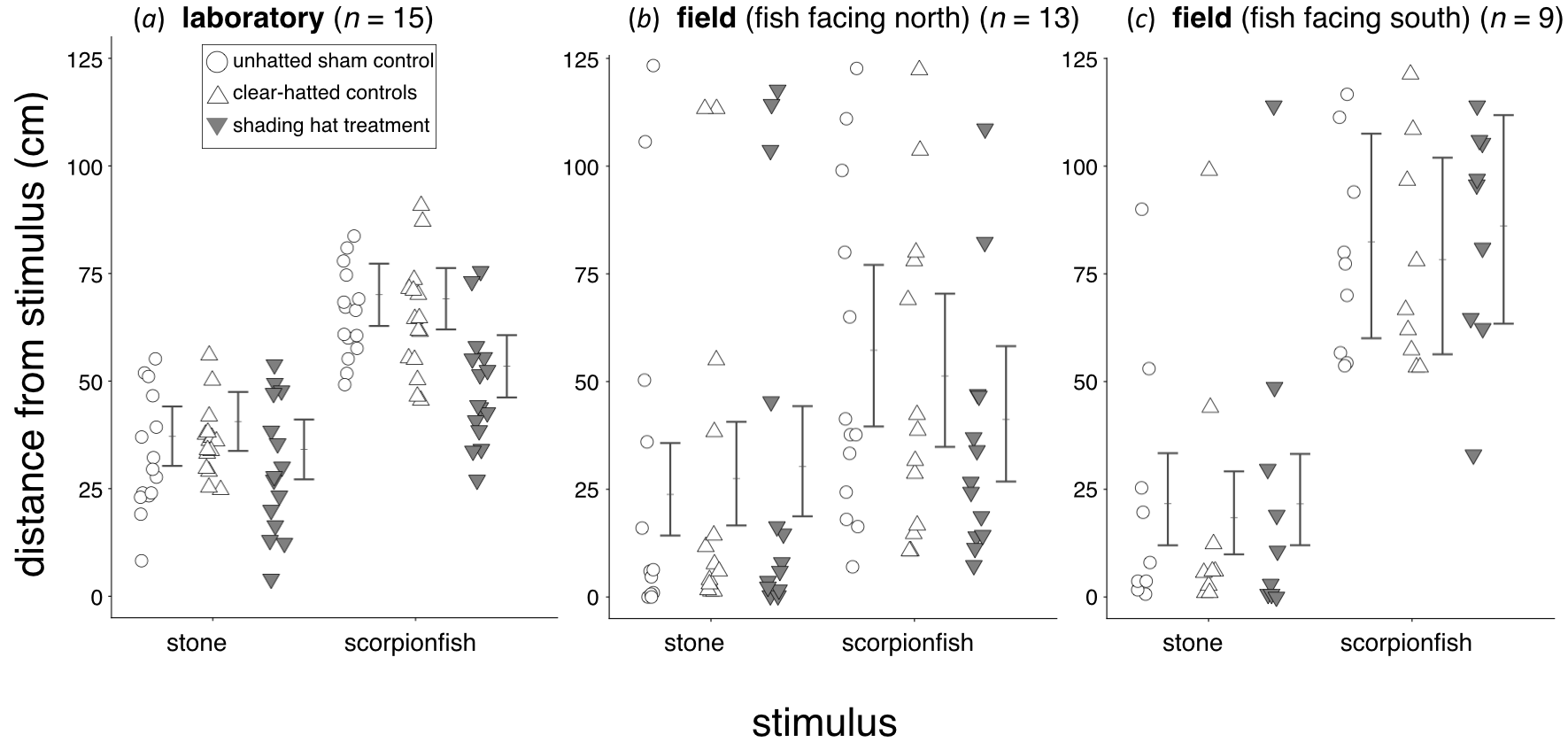
**

**Figure S2: Effect of hat treatment on the average distance from the stimulus as a function of stimulus type (stone or scorpionfish) in replicate experiments in the laboratory (*a*) and the field (*b-c*) showing** **statistical effect sizes when using complete triplets only and analysing the three treatments separately.** Not pooling the two controls limits the analysis to triplets in which no individual had lost its hat in the course of the experiment. This dramatically reduced the data set of the field experiment to *n* = 13 in fish facing north (*n* = 24 for pooled controls, see main text) and *n* = 9 to fish facing south (*n* = 19 for pooled controls, see main text). However, it also demonstrates the similarity between the two controls in their response (see ESM Table S1, S2 for statistics). Comparing *b-c* with figure 2***b-c*** in the main document shows that the variance is higher in the field trials, explaining the wider error bars. We attribute this variation to the presence of other fish outside the tank (including predators) which may have caused triplefins to respond to more than the experimental visual stimuli. By placing tanks on floats in the second field experiment, we reduced such interference. Comparing *a* with figure 2*a* in the main documents shows that the two analyses are qualitatively similar if sample size stays the same (as it is the case for the lab experiment). *Symbols*: average per individual from three positions taken up to 15-21 hours after exposure to the stimulus; error bars: model-predicted group means ± 95 % credible intervals. Note: statistical comparisons rested on connected measures *within* triplets and 5 (lab) or 3 (field) data points per stimulus, making error bars difficult to interpret as indicator of significance.

**Table S1.** **Statistical analysis of the laboratory data presented in figure 2*a*. Linear Mixed Models with distance from the two visual stimuli (scorpionfish or stone) as the response variable.** Given that the two control treatments did not differ in their response to the two stimuli (*a*), their respective measurements were averaged for the main analysis in which the response of control and shaded treatments to both stimuli was compared (*b*, figure 2*a*). The final model (*c*) tests the difference between the controls and the shaded treatment in their response to the scorpionfish only. CI = credible interval. For factorial predictors, estimates are computed using the indicated intercept levels as reference. This choice is arbitrary and does not affect overall conclusions.

|  | Predictors | Predicted mean | Lower 95% CI | Upper 95% CI | *P* |
| --- | --- | --- | --- | --- | --- |
| (*a*) Response of unhatted and clear-hatted controls to both stimuli  *n* = 15 triplets, *R*^2^_marg_ = 0.30, *R*^2^_cond_ = 0.31 | | | | | |
|  | *intercept* (stone & no hat) | 25.770 | 16.031 | 35.524 | < 0.0001 |
|  | *hat treatment* (clear hat) | 3.416 | -4.110 | 10.881 | 0.373 |
|  | *stimulus* (scorpionfish) | 32.917 | 25.385 | 40.419 | < 0.0001 |
|  | *hat treatment* x s*timulus* | -4.421 | -14.913 | 6.227 | 0.412 |
|  | *stimulus order* | 4.526 | -0.791 | 9.886 | 0.100 |
| (*b*) Response of averaged controls and shaded individuals to both stimuli  *n* = 15 triplets, *R*^2^_marg_ = 0.28, *R*^2^_cond_ = 0.28 | | | | | |
|  | *intercept* (stone & controls) | 31.994 | 27.466 | 36.667 | < 0.0001 |
|  | *hat treatment* (shading hat) | -4.849 | -11.388 | 1.683 | 0.151 |
|  | *stimulus* (scorpionfish) | 30.700 | 25.304 | 35.980 | < 0.0001 |
|  | *hat treatment* x s*timulus* | -11.390 | -20.570 | -2.142 | 0.017 |
|  | *stimulus order* | 4.580 | 0.190 | 8.936 | 0.041 |
| (*c*) Response of averaged controls and shaded individuals to the scorpionfish stimulus only  *n* = 15 triplets, *R^2^_marg_ = 0.14, R^2^_cond_ = 0.23* | | | | | |
|  | *intercept* (controls) | 62.918 | 57.127 | 68.660 | < 0.0001 |
|  | *hat treatment* (shading hat) | -16.220 | -21.417 | -11.043 | < 0.0001 |
|  | *stimulus order* | 4.256 | -4.007 | 12.420 | 0.331 |

**Table S2.** Statistical analysis of the field data presented in figure 2*b-c*. Linear Mixed Models with the distance from the two visual stimuli (scorpionfish or stone) as the response variable. Given that the two control treatments did not differ in their response to the two stimuli (*a*), the respective measurements were averaged for the main analysis that compared the response of control and shaded treatments to both stimuli split by the two orientations (*b-c*, figure 3*b-c*). Note that predicted means and their credible intervals (CI) are based on a square-root transformation of the response variable (Materials and Methods). For factorial predictors, estimates are computed using the indicated intercept levels as reference. This choice is arbitrary and does not affect conclusions. Note that the sample size for the comparison of the controls is small (*n* = 22 out of 50 tested triplets). This is the number of triplets in which all triplefins still carried their hat at the end of the experiment which is required to unequivocally assign treatments. The sample sizes for (b) and (c) are larger because all triplets in which only the clear-hatted triplefin had lost its hat could be included.

|  | Predictors | Predicted mean | Lower 95% CI | Upper 95% CI | *P* |
| --- | --- | --- | --- | --- | --- |
| (*a*) Response of unhatted and clear-hatted controls to both stimuli and orientations  *n* = 22 triplets, *R^2^*_marg_ = 0.31*, R^2^*_cond_ = 0.56 | | | | | |
|  | *intercept* (stone & no hat & facing N) | 2.323 | 0.773 | 3.864 | 0.004 |
|  | *hat treatment* (clear hat) | 0.085 | -0.779 | 0.930 | 0.844 |
|  | *stimulus* (scorpionfish) | 3.068 | 2.082 | 4.061 | < 0.0001 |
|  | *hat treatment* x s*timulus* | -0.438 | -1.667 | 0.758 | 0.476 |
|  | *orientation* (facing S) | -0.534 | -2.358 | 1.298 | 0.556 |
|  | *stimulus* x *orientation* | 2.698 | 1.422 | 3.962 | < 0.0001 |
|  | *stimulus order* | 0.895 | 0.268 | 1.528 | 0.005 |
| (*b*) North-facing triplefins  1. Response of averaged controls and shaded individuals to both stimuli  *n* = 24 triplets, *R*^2^_marg_ = 0.23, *R*^2^_cond_ = 0.45 | | | | | |
|  | *intercept* (stone & controls) | 1.501 | 0.332 | 2.686 | 0.014 |
|  | *hat treatment* (shading hat) | 0.537 | -0.282 | 1.351 | 0.201 |
|  | *stimulus* (scorpionfish) | 3.265 | 2.460 | 4.090 | < 0.0001 |
|  | *hat treatment* x *stimulus* | -1.199 | -2.337 | -0.071 | 0.038 |
|  | *stimulus order* | 1.412 | 0.827 | 2.004 | < 0.0001 |
| 2. Response of averaged controls and shaded individuals to the scorpionfish stimulus only *n* = 23 triplets, *R*^2^_marg_ = 0.03, *R*^2^_cond_ = 0.61 | | | | | |
|  | *intercept* (controls) | 6.138 | 3.681 | 8.643 | < 0.0001 |
|  | *hat treatment* (shading hat) | -0.670 | -1.185 | -0.165 | 0.011 |
|  | *stimulus order* | 0.492 | -1.157 | 2.108 | 0.551 |
| (*c*) South-facing triplefins  Comparison of averaged controls and shaded individuals to both stimuli  *n* = 19 triplets, *R^2^_marg_* = 0.40, *R^2^_cond_* = 0.58 | | | | | |
|  | *intercept* (stone & controls) | 5.208 | 3.780 | 6.610 | < 0.0001 |
|  | *hat treatment* (shading hat) | -0.890 | -1.815 | 0.034 | 0.055 |
|  | *stimulus* (scorpionfish) | 4.173 | 3.223 | 5.123 | < 0.0001 |
|  | *hat treatment* x *stimulus* | 0.771 | -0.522 | 2.108 | 0.248 |
|  | *stimulus order* | -0.513 | -1.203 | 0.179 | 0.138 |

**Table S3.** Statistical analysis of the field data presented in figure 3. Generalized Linear Mixed Model (*n* clear hat = 42, *n* shading hat = 38, *R*^2^_marg_ = 0.46) with proportional distance to the visual stimulus (scorpionfish only) as the response variable.  Note that predicted means and their credible intervals (CI) are based on a beta distribution with logit link (see Materials and Methods). For factorial predictors, estimates are computed using the indicated intercept levels as reference. This choice is arbitrary and does not affect the overall conclusions. This model includes a first-order autoregressive (AR1 = 0.86) variance structure to correct for temporal dependency in the observations of the same individuals.

| Predictors | Predicted mean | Lower 95% CI | Upper 95% CI | *P* |
| --- | --- | --- | --- | --- |
| *intercept* (clear hat) | 0.674 | 0.610 | 0.735 | < 0.0001 |
| *hat treatment* (shading hat) | -0.086 | -0.166 | -0.007 | 0.034 |
| *time* | 0.103 | 0.071 | 0.137 | < 0.0001 |
| *time^2^* | -0.043 | -0.071 | -0.013 | 0.003 |
| *hat treatment x time* | 0.052 | 0.004 | 0.099 | 0.036 |

**3. Details of the visual modelling**

### **Methods S2**

### **Spectrometric measurements and estimates of solid angles**

Spectroradiometric measurements were obtained with a calibrated SpectraScan PR-740 (PhotoResearch, New York USA) encased in an underwater housing (BS Kinetics, Germany). This device measures spectral radiance (watts sr^-1^ m^-2^ nm^-1^) of an area with defined solid angle. The downwelling light was estimated by measuring the radiance of a polytetrafluoroethylene (PTFE) diffuse white reflectance standard (Berghof Fluoroplastic Technology GmbH, Germany) positioned parallel to the water surface from a 45° angle. Radiance values were subsequently converted to photon radiance (photons s^-1^ sr^-1^ m^-2^ nm^-1^).

We determined the relationship between the radiance of the ocular spark and that of a white PTFE standard exposed to downwelling light in live triplefins. Fish mildly sedated with clove oil (*n* = 10) were placed in an aquarium illuminated with a Leica EL 6000 source and a liquid-filled light guide suspended ~ 20 cm above the tank. Spark radiance was normalized by comparing it to a white standard at 45° from normal positioned at the same location as the fish. For each fish, three measurements were obtained from each eye. Because of microscopic movements of the fish (mainly breathing) and the setup, not all measurements were perfectly centred. We therefore took the highest value for each fish relative to the standard to generate a scale of expected spark radiance for the model. Ocular sparks show little if any angular dependence in the horizontal plane, and can be therefore considered diffuse reflectors in that plane [6]. The sizes of the triplefin pupil (*n* = 35), the iridal chromatophore spot on which ocular sparks are focused (*n* = 10), and the scorpionfish pupil (*n* = 20) were measured in ImageJ [7] using scaled images. Natural baseline pupil radiance of three different scorpionfish was measured orthogonally to the pupil from the perspective of the triplefins during the second field experiment using a Photo Research PR-740 spectroradiometer.

The solid angle of the scorpionfish pupil ($\Omega_{sp}$) as perceived by the (dimensionless) center of the triplefin's pupil at distance d was estimated using the formula:

$$\Omega_{sp}=\frac{\pi r_{sp}^{2}}{d^{2}}$$

The solid angle of the ocular spark as seen from the perspective of a scorpionfish eye ($\Omega_{os}$) needs to be corrected for the fact that the ocular spark is below the triplefin's pupil by a distance Δ = 0.00109 m. The radius of the ocular spark at this distance as perceived by the scorpionfish can be calculated by multiplying the original diameter ros with the ratio of the original distance d divided by the hypotenuse of the right-angled triangle defined by Δ and d:

$$r_{os}^{'}=r_{os}\frac{d}{\sqrt{{}^{2}+d^{2}}}$$

The solid angle of the ocular park as perceived by the (dimensionless) center of the scorpionfish’s pupil can then be calculated as:

$$\Omega_{os}=\frac{\pi r_{os}^{'2}}{d^{2}}$$

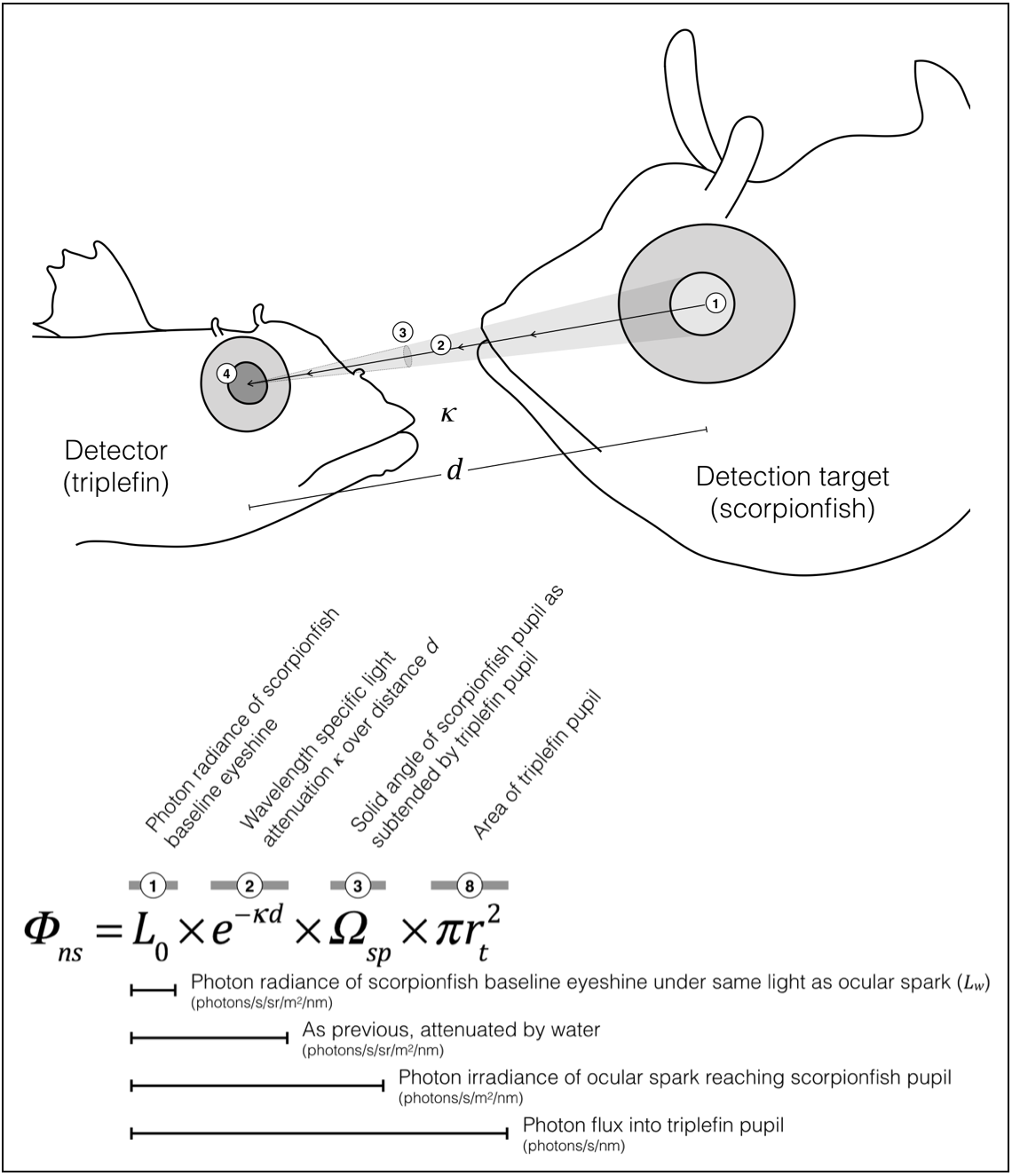


**Figure S3:** Visual representation of how the photon flux 𝜱*_ns_* originating from baseline scorpionfish eyeshine entering a triplefin's pupil is calculated. This case excludes the effect of an ocular spark, which is shown in figure S3. Baseline scorpionfish eyeshine was measured directly in the tanks at 10 m depth (second field experiment). Note that all calculations are performed for each nm (400-700 nm). See Table S4 for an explanation of symbols and indices.


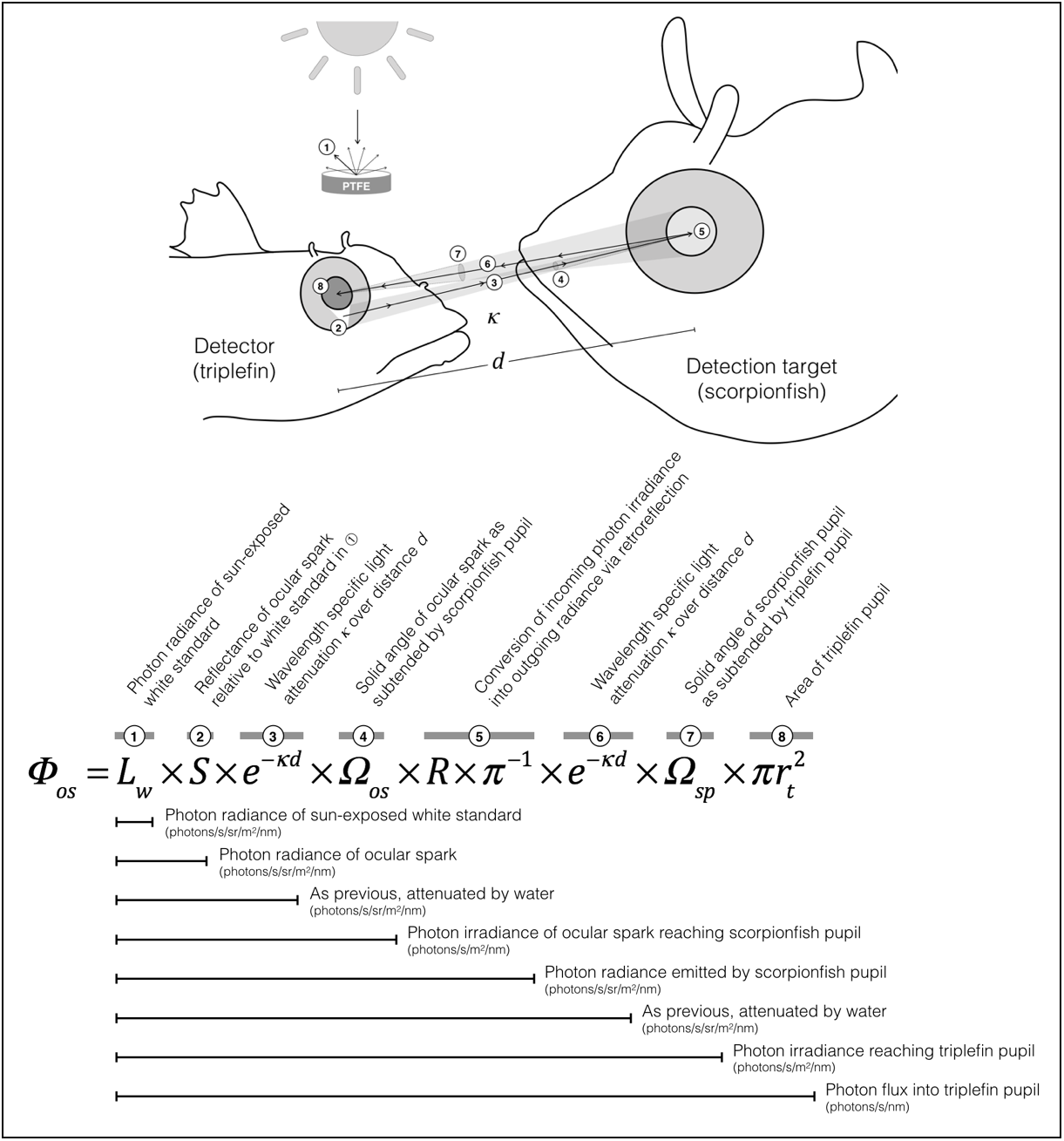


**Figure S4:** Visual representation of how much of the photon flux 𝜱*_os_* generated by a triplefin's ocular spark is reflected as scorpionfish eyeshine and ultimately reaches a triplefin's pupil. This effect needs to be added on top of baseline scorpionfish eyeshine (figure S2), to obtain the total photon flux from a scorpionfish eye reaching the eye of a triplefin with its ocular spark on. Note that all calculations are performed for each nm (400-700 nm). See Table S4 for an explanation of symbols and indices.

**Table S4.** Symbols and indices used in the equations to calculate the photon flux of the scorpionfish pupil reaching the triplefin, with and without the contribution of an ocular spark.

| **Symbol** | **Definitions and units** |
| --- | --- |
| *L* | Photon radiance (photons s^-1^ sr^-1^ m^-2^) |
| *S* | Blue ocular spark reflectance (proportion in relation to PTFE white standard) |
| *d* | Distance between triplefin and scorpionfish (m) |
| Δ | Mean displacement of ocular spark relative to triplefin pupil centre (0.00109 m) |
| *r* | Radius (m) |
| $\Omega$ | Solid angle (sr) |
| *R* | Reflectance of coaxially illuminated scorpionfish pupil  (prop. in relation to PTFE white standard) |
| *𝛋* | Diffuse attenuation coefficient (m^-1^) |
| $\Phi$ | Photon flux (photons s^-1^) |
| **Indices** | **Meaning and use** |
| *0* | Distance = 0, used in *L_0_* |
| *w* | Used for downwelling light from the water surface, used in *L_w_* |
| *ns* | Abbreviation for "no ocular spark", used in Φ*_ns_* |
| *os* | Abbreviation for ocular spark of a triplefin, used in *L_os_*, *r_os_* and Ω*_os_* |
| *sp* | Abbreviation for scorpionfish pupil, used in *L_sp_*, *r_sp_* and Ω*_sp_* |
| *t* | Abbreviation for triplefin, used in *r_t_* |

**4. References** (ESM)

[1] Schielzeth, H. & Forstmeier, W. 2009 Conclusions beyond support: overconfident estimates in mixed models. *Behav Ecol* **20**, 416-420. (doi:10.1093/beheco/arn145).

[2] Zuur, A.F., Ieno, E.N., Walker, N.J., Saveliev, A.A. & Smith, G.M. 2009 *Mixed Effects Models and Extensions in Ecology with R*, Springer, New York.

[3] Lefcheck, J.S. & Freckleton, R. 2016 piecewiseSEM: Piecewise structural equation modelling inr for ecology, evolution, and systematics. *Methods Ecol Evol* **7**, 573-579. (doi:10.1111/2041-210x.12512).

[4] Nakagawa, S. & Schielzeth, H. 2010 Repeatability for Gaussian and non-Gaussian data: a practical guide for biologists. *Biol Rev* **85**, 935-956. (doi:10.1111/j.1469-185X.2010.00141.x).

[5] Zuur, A.F., Ieno, E.N. & Freckleton, R. 2016 A protocol for conducting and presenting results of regression-type analyses. *Methods Ecol Evol* **7**, 636-645. (doi:10.1111/2041-210x.12577).

[6] Bitton, P.-P., Christmann, S.A.Y., Santon, M., Harant, U.K. & Michiels, N.K. 2019 Visual modelling supports the potential for prey detection by means of diurnal active photolocation in a small cryptobenthic fish. *Sci Rep* **9**, 8089.

[7] Abràmoff, M.D., Magalhães, P.J. & Ram, S.J. 2004 Image processing with ImageJ. *Biophotonics Int* **11**, 36-42.
